# Tryptophan Co-Metabolism at the Host-Pathogen Interface

**DOI:** 10.3389/fimmu.2020.00067

**Published:** 2020-01-29

**Authors:** Claudio Costantini, Marina M. Bellet, Giorgia Renga, Claudia Stincardini, Monica Borghi, Marilena Pariano, Barbara Cellini, Nancy Keller, Luigina Romani, Teresa Zelante

**Affiliations:** ^1^Department of Experimental Medicine, University of Perugia, Perugia, Italy; ^2^Department of Medical Microbiology and Immunology, Department of Bacteriology, University of Wisconsin-Madison, Madison, WI, United States

**Keywords:** tryptophan, co-metabolism, xenobiotic receptor, microbiota, kynurenine, 3-IAld

## Host-microbe Tryptophan Co-metabolism

Microbes have evolved to exploit humans as a rich source of nutrients to support survival and replication. Although mammals and microbes may differ in their requirement for tryptophan (Trp), being an essential amino acid in the former and produced, with some exceptions, by bacteria and fungi, common catabolic enzymes are shared by both host and pathogens. Indoleamine 2,3-dioxygenases (IDOs) catabolize Trp to kynurenines and are widely distributed from bacteria to metazoans. The evolutionary conservation of the kynurenine pathway may be linked to the importance of the *de novo* synthesis of nicotinamide adenine dinucleotide (NAD+), to which it ultimately leads, although additional functions of kynurenines are increasingly being recognized. Indeed, it is now clearly established that mammalian IDOs regulate infection and drive immune tolerance by means of Trp deprivation and the generation of active metabolites, including kynurenines. An additional level of complexity can be envisaged when microbes utilize Trp *via* alternative pathways upon colonization of the host in a relationship that can be either commensalism or pathogenic. In these situations, the host and microbes are found to share common substrates but the presence of dissimilar metabolic pathways may result in the generation of metabolites, such as indoles or tryptamine that can cross-regulate each others metabolism. Here, we discuss the potential relevance of Co-Trp metabolism or alternative secondary pathways of Trp degradation in modulating host immune response and eventually the xenobiotic receptors (XRs), while regulating microbe fitness. These concepts are expected to open a novel scenario in which a comprehensive assessment of the metabolic status is crucial to correctly evaluate pathological colonization and drive the most appropriate therapeutic strategy.

## Co-metabolism Dictates Pathogen Virulence

Trp is one of the 20 amino acids used for building proteins with the unique characteristic of bearing an indole, a bicyclic ring formed by a benzene and a pyrrole group, linked to the α-carbon by a –CH2-group (1). The presence of the indole group not only dictates the biochemical properties of Trp, a highly hydrophobic amino acid that guarantees the stabilization of protein and peptide structures, but also makes Trp a reservoir of indole-based bioactive molecules with fundamental implications in organism physiopathology (1, 2). The relevance of Trp and its catabolic pathways acquires a novel dimension when they are envisioned in the context of a relationship between host and microbes. Indeed, the different entities involved in the relationship share the same Trp substrate, which is fundamental for all the parties at play, but is catabolized along peculiar patterns. Therefore, multiple levels of interactions can be foreseen, starting from the competition of the Trp substrate to the generation of bioactive molecules *via* shared or exclusive catabolic pathways with cross-regulatory properties, and each will be discussed upon in the following sections.

First, the requirement for Trp differs between the organisms. Indeed, while Trp is an essential amino acid in mammals, microorganisms, and higher plants possess the ability to synthesize Trp from chorismate, a common precursor of aromatic amino acids produced by the shikimate pathway from phosphoenol pyruvate and erythrose-4-phosphate (3). This dependence of mammals from external sources of Trp creates a first level of interaction in the host/microbe interface. Indeed, mammals might obtain Trp not only from the diet, but also from commensal microorganisms that possess the shikimate pathway and may represent a source of Trp. On the contrary, pathogens may co-opt host mechanisms of Trp degradation as a strategy to evade the host immune response (4). For instance, in a murine model, the gut pathogen *Clostridium difficile* induced IDO1 expression to deplete the Trp pool and increase kynurenine production in the cecal tissue by IDO1-expressing CD11c^+^ myeloid cells, among other stromal cells. As a consequence, neutrophil accumulation and pathogen clearance were limited (4, 5). Other pathogens however, depend on the host for Trp availability, including common intracellular pathogens, and it is the host that depletes the Trp pool to limit the virulence of the pathogens (4, 6). For instance, the parasite *Toxoplasma gondii* is Trp-auxotroph and IDO activity suppresses its growth, as first demonstrated by Pfefferkorn and co-workers in cultured human fibroblasts treated with IFNγ (7). Thus, Trp itself appears as a double-edged sword in the host-microbe interaction: on the one hand, it may ensure a positive symbioses between the host and Trp synthesizing microbes; on the other hand, it may be used as a weapon to deprive the host or, vice versa, Trp-auxotroph pathogens of Trp, resulting in increased or reduced virulence, respectively.

Second, Trp may be catabolized via shared catabolic pathways. In mammals, Trp is metabolized along four different pathways leading to the formation of (i) serotonin and melatonin, (ii) tryptamine, (iii) indolepyruvic acid, and (iv) kynurenine (8). The kynurenine pathway accounts for nearly 95% of all Trp degradation (8), and the rate-limiting step is catalyzed by one of three enzymes, namely indoleamine 2,3-dioxygenase 1 (IDO1), IDO2, and tryptophan 2,3-dioxygenase (TDO), with distinct localization, affinity, and regulation (8). The evolution of IDOs and TDO has been the subject of intense research. TDO is widely present in metazoan and many bacterial species, but not in fungi, and is characterized by a high efficiency for Trp degradation throughout the evolution (9). On the contrary, IDO is found in mammals, lower vertebrates, invertebrates, fungi, and bacterial species, but only mammalian IDO1 and fungal IDOs show high efficiency for Trp degradation (9). Irrespective of the ancestral role of IDOs and TDO and their evolution, it is evident that microorganisms are endowed with the ability to catabolize Trp along the kynurenine pathway, as partially demonstrated by experiments in germ-free mice that show a decrease in the kynurenine pathway (10). In this scenario, two opposite outcomes are possible. Indeed, not only the distinct dioxygenases might compete for the same substrate to produce kynurenine for self-advantage, but they can also compensate each other in physiological or pathological conditions characterized by kynurenine deficiency, again identifying Trp and its catabolism as a double-edged sword in host-microbe interaction. Unfortunately, studies on the kynurenine pathway in microbes and how it intersects with host metabolism are still very scarce, and it is not possible to draw any conclusions in support of one or the other possibility.

As a third level of interaction, Trp may be catalyzed via distinct catabolic pathways by the host and microbes, resulting in the generation of metabolites that can cross-regulate each other metabolism. In recent years, we have identified and characterized the “postbiotic” molecule indole-3-aldehyde (3-IAld) derived from the microbial degradation of Trp and produced by probiotics such as lactobacilli (11). 3-IAld proved critical in the maintenance and restoration of intestinal epithelial integrity. Indeed, by binding the aryl hydrocarbon receptor (AhR) and activating the expression of IL-22, 3-IAld promotes the repair of the intestinal epithelial lining and the reduction of inflammatory markers (11). Another interesting example is represented by indole, produced by bacteria and some plants from Trp *via* the enzymes tryptophanase and indole-3-glycerol phosphate lyases, respectively (12). Interestingly, indole negatively regulates the virulence of various pathogens, such as the gastrointestinal tract pathogens enterohemorrhagic *Escherichia coli* (EHEC) (13, 14) and *Citrobacter rodentium* (14). For instance, mice infected with *C. rodentium* and manipulated to contain different concentrations of indole in the gastrointestinal tract showed an inverse correlation between colonization/mortality and amounts of indole (14). In addition, indole can enhance the competitiveness of commensal microorganisms, for instance by promoting the growth of *E. coli* in mixed-cultures with *Pseudomonas aeruginosa* via inhibition of quorum sensing (15). However, microorganisms may also adopt specialized ways to use Trp and catabolic intermediates as pathogenic molecules. For instance, *A. fumigatus* can incorporate Trp and/or anthranilate *via* non-ribosomal peptide synthetases to generate toxic molecules (16), such as the Trp-derived iron (III)-complex hexadehydroastechrome that increased the virulence of *A. fumigatus* and the mortality in a neutropenic murine pulmonary model upon overexpression (17).

Overall, these examples illustrate how Trp and catabolic molecules play a fundamental role in regulating the interaction between the host and the microbes, which can occur at multiple levels and with opposite outcomes. Indeed, Trp and its metabolites may serve to establish a symbiotic relationship or otherwise be used to weaken the partner by depleting essential molecules or creating toxic substances.

## Tryptophan Degradation by Microbes: Toxicity vs. Immunomodulation

### Indole: The Interkingdom Molecule

The enzyme tryptophanase (TnA) is responsible of Trp degradation and release of indole and indole-derivatives. Importantly, TnA is widely expressed in Gram-negative as well as in Gram-positive bacteria (14, 18). The indole ring is found in humans as the nucleus of human hormones as serotonin or melatonin, but is also found in plants in auxins, which may affect plant orientation by promoting cell division to one side of the plant in response to sunlight and gravity. Indole is also synthesized in the bowel by microbes, regulating the biofilm as quorum sensing molecule or the intestinal physiology. Thus, the indole ring being diffused in different ecosystems, is considered a type of “*archetypical hormone*” able to regulate the relation between the host and microbes in plants but also in the animal kingdom. The mechanisms of action in the host by indole and indole derivatives are not well-characterized yet, although a very large part of research has focused the mechanistic function on their capacity to bid the XRs (11, 19, 20). Interestingly, animals can't synthesize indole, while many bacteria and also fungi produce indole and indole-derivatives with some pathogens such as *A. fumigatus* synthesizing toxic indole alkaloids (21). In addition, indole and indole-derivatives are detected in the human blood as well as in peripheral tissues in physiological conditions. Frequently, indoles can be measured in urine but traces are also present in lymph nodes (22–24). A good example of co-metabolism with the host among the indole-derivatives is the indoxyl sulfate, typically representing a uremic toxin, derived from indole in the liver via the actions of cytochrome P450 enzymes (25).

### The Indole Pyruvate (IPyA) Route: A Direct Effect on Immunity

**Figure 1:**
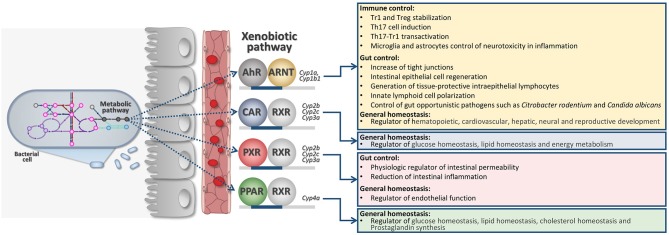
Intricate microbiota-derived metabolic pathways may affect host XR activation and general host physiology. AhR, Aryl hydrocarbon receptor; ARNT, Aryl Hydrocarbon Receptor Nuclear Translocator; CAR, Constitutive Androstane Receptor; RXR, Retinoid X Receptor; PXR, Pregnane X Receptor; PPAR, Peroxisome Proliferator-Activated Receptors.

The indole-3-pyruvic acid (IPyA) route is key to converting aromatic amino acids to aroma compounds via transamination of Trp (26). IPyA secondary metabolites are indol-3-acetaldehyde (3-IAAld), indole-3-acetic-acid (IAA), and indole-3-carboxaldehyde (3-IAld)—all known as being AhR ligands (11) (Figure 1). AhR is part of the XRs family. XRs have evolved as cellular sensors for ligands (endogenous and exogenous) able to transcribe for genes encoding for drug-metabolizing enzymes.

XRs are also extremely involved in regulating general physiology since they can also transcribe for genes involved in immune regulation, cell metabolism, energy homeostasis (Figure 1). Families of XRs may bind a very large family of unrelated ligands by direct or indirect binding (27, 28). XRs may include the pregnane X receptor (PXR), the AhR, the constitutive androstane receptor (CAR), and the peroxisome proliferator-activated receptors (PPARs) (Figure 1). More recently, XRs family has been deeply investigated for their ability to directly communicate with the gut microbiota (11, 29, 30).

XRs respond to different metabolites produced by the host as well as by the microbiota. Thus, metabolism and co-metabolism are strictly related to XR activation pathway (30). Therefore, the acute or chronic symptoms of dysbacteriosis generally reflect the XR role in regulating the host physiology as energy metabolism, glucose homeostasis, immune-regulation.

Indeed, it was demonstrated that certain bacterial tryptophan-derived metabolites activate PXR particularly expressed in intestinal epithelial cells. The downstream signaling of intestinal PXR affects murine intestinal permeability, gut inflammation, and in peripheral tissue bile acid metabolism and drug resistance. Thus, in case of dysbiosis, where there is a severe lack of PXR ligands homeostasis is seriously compromised. Restoration of PXR signaling by using gnotobiotic mice or by administering PXR ligands, may result in abolishing pro-inflammatory signs and loss of barrier dysfunction in the context of intestinal inflammation (31).

In addition, we have demonstrated in mice that the indole-derivative 3-IAld is an AhR ligand that promotes IL-22 production. 3-IAld restored antifungal resistance and increased IL-22 production, ameliorated colitis via gut NKp46^+^ cells and via the XR AhR. These results suggest that the activity of 3-IAld could be exploited to guarantee homeostasis and microbial cooperation at mucosal surfaces in conditions of immune dysregulation (11, 32). More recently, 3-IAld has been proved also changing together with a tryptophan-rich diet, the program of intraepithelial CD4^+^ T cells into immunoregulatory T cells in mice (20).

### Kynurenines: Immunomodulatory Functions

As in mammalians, Trp may also be degraded in to kynurenines by microbes. For example, *Pseudomonas aeruginosa*, a Gram-negative bacteria frequently involved in healthcare-associated pneumonia, catabolizes tryptophan through the kynurenine pathway. Thus, bacterial metabolites may interfere with the host's immune response during *in vivo* infection and acute lung injury (33). Another important evidence of co-metabolism, was demonstrated for the opportunistic fungus *Candida albicans* (34). Trp metabolites produced by the fungus *Candida*, in particular 5-hydroxytryptophan metabolites, are also able to modulate Th17 response, similarly to kynurenines as shown for mammalians (34). The opportunistic fungal pathogen *A. fumigatus* also has three *ido* genes (*idoA,B,C*) in its genome (35–37). Enzymatic studies suggest that Idos of *A. oryzae*, participate in Trp degradation (38). Furthermore, previous studies on *A. fumigatus* grown on Trp showed upregulation of these *ido* genes (36). However, the relative contributions of individual Idos and adaptation to the host's environment, as well as the impact of kynurenines released by the fungus during lung infection *in vivo*, remain unclear.

## Conclusions

The scientific proof for a contribution of the XRs in the control of barrier function and immune regulation would serve as a basis toward improvement of non-toxic probes and ligands as drugs (30). This is also put forward by the fact that several of those microbial metabolites are found in human blood at levels comparable to host metabolites (3-IAld 0.01–0.1 μM; IAA 0.1–1 μM), suggesting that systemic responses may be easily activated by targeted XR-based therapy. These systems are thought to provide an additional level of interchange between the microbes and the host at the edge of their co-metabolism (Figure 1).

## Author Contributions

CC and TZ wrote the manuscript. MMB, GR, CS, MB, MP, BC, NK, and LR edited the manuscript and provided valuable discussions and criticisms.

### Conflict of Interest

The authors declare that the research was conducted in the absence of any commercial or financial relationships that could be construed as a potential conflict of interest.
